# The heart’s eye: how mental imagery influences romantic emotion

**DOI:** 10.3389/fpsyg.2025.1608874

**Published:** 2025-09-17

**Authors:** Boran Cui, Yulin Kong, Weibo Zhang

**Affiliations:** ^1^Dalton Academy, The Affiliated High School of Peking University, Beijing, China; ^2^Moonshot Academy, Beijing, China

**Keywords:** mental imagery, aphantasia, romantic emotion, neural correlates, autonomic responses, EEG, HRV

## Abstract

**Introduction:**

While mental imagery—the capacity to generate perceptual-like experiences in the absence of external stimuli—has been studied in fear and other domains, its influence in romantic emotional experiences has not been directly examined. Based on this hypothesis, we investigated how imagery vividness influences romantic emotions and their physiological underpinnings.

**Methods:**

Firstly, we reviewed our previous questionnaire data. Furthermore, we compared individuals with vivid imagery and aphantasia, a condition characterized by the absence of voluntary visual imagery, using electroencephalogram (EEG) and heart rate variability (HRV) during a romantic imagery task.

**Results:**

Those with vivid imagery showed stronger neural markers (larger P3 amplitudes, extended LPPs, reduced occipital alpha activity) and heightened autonomic arousal (increased heart rate, suppressed HRV). Aphantasic participants exhibited muted neural responses and minimal autonomic changes, reflecting weaker emotional embodiment.

**Discussion:**

These findings underscore that vivid visual imagery is a crucial driver of romantic emotional intensity and duration, whereas the absence of imagery can lead to a markedly diminished emotional experience.

## 1 Introduction

Mental imagery is the process of generating perceptual-like experiences in the absence of external stimuli—essentially ‘seeing with the mind’s eye’, and imagery ability (or vividness) varies widely between individuals (Ji et al., 2016; Boccaccio et al., 2024). Since Galton’s 19th-century surveys of visualization ability (Galton, 1880), research (Jin et al., 2024) has recognized that people vary widely in imagery vividness. Visual imagery is a fundamental cognitive process linked to memory, creativity, and emotion (Holmes and Mathews, 2010). Crucially, mental images can evoke emotions almost as powerfully as actual stimuli: imagery engages emotional brain circuits, prompting responses “as if” the imagined content were actual (Ji et al., 2016). Holmes and Mathews (2010) demonstrated that vividly imagining emotional events produces stronger affective reactions than verbal thinking about the same events. In other words, vividly picturing a loved one’s embrace or a romantic encounter can engage the same autonomic and neural systems that would activate during the actual experience.

The term *aphantasia* was introduced to describe the inability to generate visual images (Zeman et al., 2015). These individuals lead otherwise normal lives but report that the “mind’s eye” is essentially blind—they cannot visualize familiar faces or scenes even when they try (Keogh and Pearson, 2018). This condition provides a natural experiment for understanding how imagery contributes to emotion and desire. If mental imagery truly amplifies emotions, one would predict that aphantasic individuals might experience emotional situations differently, especially scenarios that typically rely on visualization (e.g., reminiscing about a partner’s face or fantasizing about an intimate moment). Indeed, emerging evidence suggests aphantasia can dampen certain emotional responses. Aphantasic individuals report reduced emotional engagement and empathy when reading descriptive stories, and they show markedly lower fear responses to scary narratives compared to individuals with vivid imagery (Wicken et al., 2021). Similarly, aphantasia has been linked to blunted emotional responses to music, consistent with the idea that vivid imagery makes thoughts “come alive,” recruiting emotional circuitry and intensifying feelings. In general, vivid imagers tend to feel greater empathy and emotional impact from imagined scenarios, whereas those without imagery may rely more on external cues to generate emotion.

Romantic relationships partly thrive on rich cognitive and emotional simulations—daydreaming about a loved one, visualizing future interactions, or recalling past intimate moments. Such imagery can evoke strong affectionate feelings and physiological arousal, essentially “rehearsing” attachment bonds in the mind. Neuroimaging studies of love have shown that thinking about one’s beloved activates brain regions tied to reward, attachment, and emotion (e.g., dopaminergic midbrain, ventral striatum, anterior cingulate, and insula; Aron et al., 2005). Early-stage romantic love induces measurable neurochemical and physiological changes: for instance, new lovers exhibit elevated cortisol and altered serotonin levels, and their brains show increased activity in oxytocin-and dopamine-rich regions when viewing or imagining the partner (Fisher et al., 2006). These reactions underscore the emotional force of romantic thought. But does the strength of such love-induced responses depend on one’s imagery capacity? If one person can vividly picture their partner’s smile or an intimate evening, whereas another (with aphantasia) can only recall facts or conceptual knowledge of the partner, their emotional and bodily responses to “imagined” romantic scenarios might differ. Prior work on sexual fantasy supports this idea: individuals with more vivid imagery achieve higher subjective and physiological arousal during imagined erotic scenarios, whereas arousal in response to actual erotic stimuli (e.g., videos) is less dependent on imagery ability. In one study, vividness of visual imagery (measured by the Vividness of Visual Imagery Questionnaire, VVIQ) significantly predicted both the self-reported intensity of sexual arousal and objective measures (e.g., penile engorgement) when participants engaged in guided sexual fantasy. By contrast, when viewing real erotic films, participants’ arousal did *not* correlate with their imagery vividness, indicating that mental imagery ability specifically modulates how vividly one can internally simulate and experience a desired scenario (Smith and Over, 1987). These findings align with the suggestion that the “mind’s eye” can drive emotional experience: vividly simulating a positive encounter can elicit joy or longing, whereas a lack of imagery (e.g., aphantasia) might leave the same scenario emotionally flat.

Given this background, we hypothesized that imagery ability is a key factor in romantic emotional processing. Accordingly, before the present study we conducted a questionnaire experiment surveying 218 young adults (18–22 yrs) using VVIQ (Marks, 1973) alongside custom items probing romantic-feeling reactivity, durability, and forgetfulness (Cui et al., 2023). Participants rated how readily they developed crushes (1–10 scale), how long such feelings typically lasted, and whether attraction quickly faded when the person was out of sight. Six respondents (2.7%) met the ≤ 32 VVIQ cutoff for aphantasia, allowing us to test imagery-absence effects within the same cohort. Demographic and health screeners excluded visual or neurological confounds, and attention checks ensured data integrity.

Analyses revealed that imagery vividness was orthogonal to the onset of attraction (*r* ≈ 0.08, n.s.) yet reliably predicted its persistence: higher VVIQ scores correlated with sustaining romantic feelings (*r* ≈ 0.20; *p* < 0.005) and resisting rapid forgetting (*r* ≈ −0.20; *p* < 0.005), even after Holm–Bonferroni correction (Cui et al., 2023). Conversely, participants meeting the aphantasia criterion were over-represented among those whose interest evaporated within days. These findings position mental imagery not as the spark that ignites desire but as the cognitive “fuel” that keeps it burning—clarifying that vivid internal representations help romance endure once external cues disappear and setting the stage for our subsequent neural and autonomic investigations into this imagery–desire linkage.

In this research, we conducted a physiological experiment to examine individuals at the extremes of imagery vividness—those with richly detailed imagery and those with aphantasia—during a controlled romantic-imagery task. High-density EEG and heart rate were recorded as participants pictured emotionally salient romantic scenes. In the visual domain, we analyzed time-frequency oscillations over occipital cortex, a canonical index of the sensory strength of mental images. In the affective–cognitive domain, we focused on two well-characterized event-related potentials: the P3, a centro-parietal component that indexes the allocation of attention, and the late positive potential (LPP), a sustained positivity whose amplitude scales with emotional arousal. Autonomic engagement was quantified by HRV suppression—i.e., transient vagal withdrawal—during imagery and the subsequent rebound, an established marker of sympathetic arousal (Lu et al., 2024). We predicted that high-vividness participants would show stronger occipital alpha–beta desynchronisation, larger P3 and LPP amplitudes, and greater HRV suppression, reflecting deeper perceptual and emotional immersion in the imagined romance. Conversely, we expected that aphantasic participants would display attenuated visual oscillatory changes, reduced ERP amplitudes, and minimal HRV modulation, consistent with their phenomenological reports of dim or absent imagery. By integrating neural and autonomic indices, the study seeks to clarify how the vividness of the mind’s eye shapes the experiential landscape of love and desire.

## 2 Methods and procedure

Fifty healthy adults (aged 18–30, 24 female) were selected in the study. Participants were prescreened for visual imagery ability using the Vividness of Visual Imagery Questionnaire (VVIQ). We recruited two groups: 25 individuals with high imagery vividness (VVIQ scores in the top range), 25 individuals with aphantasia (very low imagery vividness, VVIQ ≤ 32). All participants had normal or corrected-to-normal vision and no history of neurological or psychiatric disorders.

During recruitment, we gathered each participant’s background information (e.g., whether they were in a relationship, how many times they had been in love, gender, and sexual orientation), aiming to keep these factors balanced across groups to reduce their potential impact on engagement in the romantic-imagination task. Any remaining differences were then included as covariates in subsequent analyses, confirming that they did not significantly interfere with the main results. Moreover, for participants who had never experienced a romantic relationship or were unfamiliar with romantic concepts, we provided a brief explanation beforehand to ensure they understood and could immerse themselves in the imagined scenario. Nonetheless, it should be noted that their emotional responses may differ from those of participants with prior romantic experience, which constitutes a potential limitation of the study. The groups were matched for age, sex, and education. Each participant gave written informed consent.

### 2.1 Procedure and task

Participants attended a single laboratory session. After electrode placement, they were seated in a sound-attenuated, dimly lit room. We first obtained baseline recordings for EEG and cardiovascular measures (3 mins of relaxed wakefulness, eyes closed, thinking of neutral thoughts). Next, participants performed a guided romantic imagery task designed to elicit emotional and physiological responses. In this task, participants heard a series of standardized audio prompts describing a romantic scenario (for example: “*Imagine you are meeting your partner after a long time apart; you see their face light up*, *you feel their arms wrap around you*.”). The prompts were developed to be emotionally engaging and to require visualization of a loved-one or an affective interaction, without explicit sexual content. Each trial began with a short cue to prepare (a fixation cross on screen and a low tone). Then the narrative prompt (2–3 s seconds) was played, during which participants were instructed to close their eyes and vividly imagine the scenario as if it were happening. They were encouraged to immerse themselves in the feelings and to form mental images of the scene. A total of 40 trials were presented each trial lasting ~100 s and featuring a randomly selected romantic theme (e.g., reunion hug, candlelit dinner, comforting a partner, etc.), presented in counterbalanced order. This increased number of trials was chosen to improve the reliability of the EEG measures. All artifact-free trials were included in analysis: participants retained ≥ 30 clean trials, which were then averaged to compute each individual’s ERP waveform (Participants completed additional trials as needed to ensure at least 30 artifact-free epochs per person). Immediately after the imagery task, we administered a brief manipulation check: participants rated the vividness of the images they had tried to generate. As expected, high-imagery participants reported clear mental pictures, whereas low-imagery participants reported minimal or absent imagery, confirming that the intended group difference was present during the task. We note, however, that these retrospective self-reports provide only an initial validation and should be complemented by online behavioral or neural markers in future work.

In each 100-s trial of the romantic scenario, we first played a 2–3 s audio prompt to help participants enter the imagined context. After the audio ended, participants kept their eyes closed and continued to imagine the scenario until the 100-s period elapsed. Because the study primarily focused on the early neural responses to stimulus onset (i.e., the beginning of the audio prompt) during the imaginative process, we segmented the EEG data from 0 to 1,000 ms (after observing the initial EEG activation) and conducted an ERP analysis (P3 and early LPP) to capture the initial allocation of attention and emotional processing elicited by the prompt. For longer durations (> 1 s), which involved more advanced cognitive aspects, we did not provide further discussion here.

### 2.2 Physiological recording and data acquisition

**EEG recording**: EEG was recorded from 32 Ag/AgCl electrodes placed according to the 10–20 system (EMOTIV Flex 32 Channel). Signals were sampled at 128 SPS (1024 Hz internal) and referenced online to the Cz electrode, with impedances kept below 10 kΩ. Vertical and horizontal electrooculogram (EOG) channels monitored eye blinks and movements. During the task, event markers were sent to the EEG recording at critical time points: prompt onset (to time-lock imagery onset) and prompt end. These markers enabled extraction of stimulus-locked ERPs and analysis of neural activity specifically during the imagery epoch.

**ECG and HRV recording**: Heart activity was recorded via a three-lead ECG (electrodes on right clavicle, lower left rib, and lower right rib as ground) at 1000 Hz. This high sampling rate allowed precise detection of R-waves (heartbeats). The ECG trace was continually monitored, and events (such as trial start/end) were also marked in the cardiac data stream for alignment with imagery periods. From the ECG, we derived beat-to-beat intervals (RR intervals) and computed heart rate variability (HRV). We focused on the high-frequency HRV component (0.15–0.40 Hz), also known as respiratory sinus arrhythmia, which reflects parasympathetic (vagal) activity (Schneiderman et al., 2011). A decrease in high-frequency HRV indicates withdrawal of vagal tone (often accompanying emotional arousal or stress), whereas recovery of HRV after arousal reflects resumption of parasympathetic control.

## 3 Data analysis

**ERP analysis**: EEG data were band-pass filtered (0.1–30 Hz) and segmented into epochs time-locked to the onset of the imagery prompt (from *−*200 ms pre-stimulus to 1,000 ms post-onset). Trials with excessive EOG artifacts or noise were rejected, and minor blink artifacts were corrected via independent component analysis. Given the increased number of trials, each participant retained a high number of clean epochs (minimum 30), which were averaged to obtain reliable ERPs. We focused on two components: (1) the P3, a positive-going wave around 300–400 ms post-prompt, typically maximal at midline parietal sites (Pz), associated with attention and context updating (Donchin and Coles, 1988); and (2) the late positive potential (LPP), a sustained positivity over centro-parietal electrodes peaking around 500–800 ms, linked to emotional processing and imagery maintenance (D’Angiulli et al., 2021; Farah et al., 1989). For each participant, P3 amplitude was measured as the mean voltage in a 300–400 ms window at Pz (relative to a *−* 200 ms baseline). LPP amplitude was quantified as mean voltage in a later window (400–800 ms) at a pooled parietal-occipital region (Pz/POz and neighboring electrodes), and LPP *duration* was operationalized as the time (ms) from stimulus onset until the ERP waveform returned to baseline (0 *μ*V) after the peak. These metrics were then compared between groups. Within the 0–1,000 ms window, we define 400–800 ms as the typical LPP time window to compare the early emotional processing of the prompt stimulus between high-and low-imagery groups. In this study, the LPP lasted until about 800–1,000 ms before gradually returning to baseline; however, this does not imply that the entire 100-s imagination period maintains the same LPP pattern.

**Time–frequency EEG analysis**: To capture induced oscillatory activity during imagery (which can be missed by averaging in ERPs), we conducted time–frequency analyses using Morlet wavelet decomposition. For each trial, EEG data were convolved with wavelets (6-cycle) to estimate power in frequency bands from 4 to 30 Hz, spanning theta (4–7 Hz), alpha (8–13 Hz), and beta (14–30 Hz) frequencies. Power change relative to a pre-stimulus baseline was calculated. We were particularly interested in alpha-band suppression over visual cortex (occipital electrodes O1/O2 and Oz) during imagery, as alpha power decreases are a known marker of visual cortical activation (akin to opening one’s eyes or visual attention directed internally; Jin et al., 2024). We also examined frontal midline theta (associated with mental effort and scene construction) and beta changes in parietal regions (which might reflect imagery elaboration or emotional arousal). For statistical analysis, we averaged the percent change in power in these regions of interest during the imagery period (e.g., 0–1,000 ms) for each participant.

**HRV analysis**: R-wave detection was performed on the ECG to generate a continuous series of RR intervals (heart periods). We applied artifact correction (removing ectopic beats) and then resampled the interbeat interval time series at 4 Hz. For each trial, we computed time-domain HRV (root mean square of successive differences, RMSSD) and frequency-domain HRV via fast Fourier transform. High-frequency (HF) HRV power (0.15–0.4 Hz) was extracted as an index of parasympathetic activity, and low-frequency (LF, 0.04–0.15 Hz) power as a mixed sympathetic-parasympathetic index. We focused on changes in HF-HRV from baseline: a drop in HF power during imagery indicates vagal withdrawal (increased sympathetic dominance), whereas recovery is seen as HF power rising back toward baseline after the imagery ends (Schneiderman et al., 2011). For completeness, heart rate (in beats per minute) was also measured continuously and averaged over the same periods.

**Statistical analysis**: We used a combination of frequentist and Bayesian statistical approaches. Repeated-measures ANOVAs were conducted with Group (High-vividness, Aphantasia) as a between-subjects factor. For ERP components, we analyzed P3 amplitude and LPP measures with Group as a factor; for time-frequency data, we analyzed mean alpha suppression and frontal theta power change with Group; for autonomic data, we analyzed HRV suppression (imagery vs. baseline) with Group and Time (during vs. post-imagery) factors. Where appropriate, Greenhouse–Geisser corrections were applied for sphericity. In addition, we report partial *η*^2^ for effect sizes. Bayesian ANOVAs (using JASP 0.16) were performed in parallel to quantify evidence for group differences, with Bayes Factors (BF_10_) indicating how much more likely the data are under the hypothesis of a group effect versus no effect. A BF_10_ *>* 3 was considered moderate evidence, and *>* 10 strong evidence, for a group difference. Pearson correlations assessed the relationship between imagery vividness scores (as a continuous measure) and physiological responses across all individuals. We controlled the family-wise error rate at *α* = 0.05, applying Bonferroni corrections where required (α = 0.0125 for the four primary EEG contrasts; α = 0.010 for the five planned HRV tests). All data passed checks for normality and variance homogeneity; when assumptions were violated, nonparametric tests confirmed the robustness of results.

## 4 Results

### 4.1 ERP findings: P3 and late positive potential

**Figure 1:**
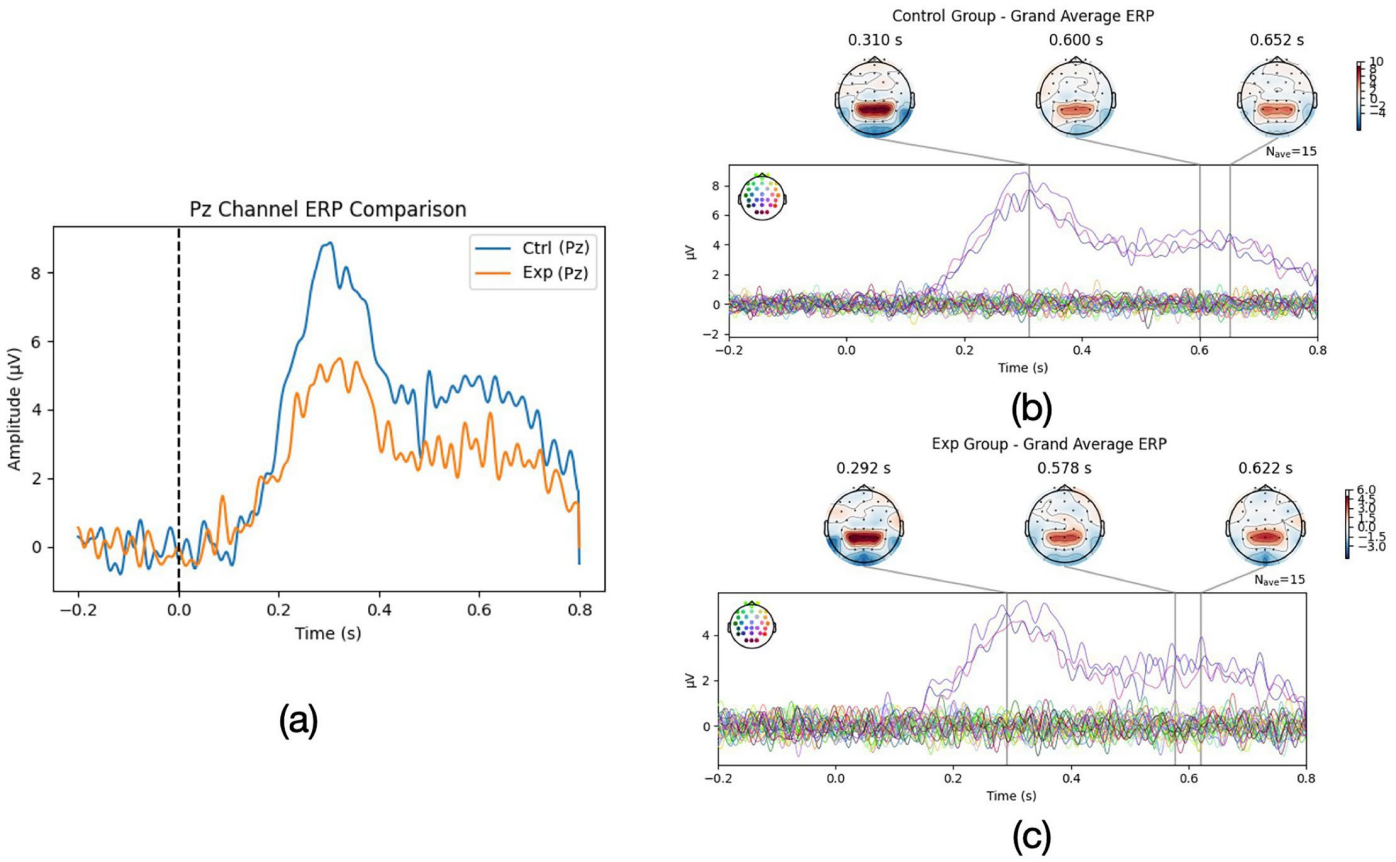
**(a)** ERP comparison of Pz channel for Exp group (aphantasia group) and Ctrl group (normal mental imagery group) and **(b,c)** whole ERP wave comparison for Exp and Ctrl group.

Figure 1a illustrates the grand-average ERP waveforms at the midline parietal electrode (Pz) for the two groups during the romantic imagery task. Both groups exhibited a discernible P3 component peaking around 320 ms after the prompt onset, followed by a prolonged positive drift (LPP) extending several hundred milliseconds. However, clear amplitude and temporal differences were present between high and low imagery individuals. The High-Vividness group (Figure 1b) showed a markedly larger P3 amplitude (mean +7.8 *±* 1.6 *μ*V) compared to the Aphantasia group (mean +4.5 *±* 1.4 *μ*V), as quantified over the 300–400 ms window. An ANOVA confirmed a significant main effect of Group on P3 amplitude [*p <* 0.001, *η*^2^ = 0.55]. Bayesian analysis strongly supported this difference (BF_10_ *>* 100).

Topographic maps (Figures 1b,c) revealed that in high imagers, the P3 was broadly distributed with a parieto-central maximum, whereas in aphantasics the positivity was weaker and more confined. This suggests that vividly imagining the scenario engaged more attention/context updating processes at stimulus onset (D’Angiulli et al., 2021), consistent with a greater mobilization of cognitive resources when an internal image was successfully generated.

Beyond the P3, group differences were even more pronounced in the later sustained positivity. In high imagery participants, the ERP remained elevated for an extended duration: their waveforms showed a classic LPP that persisted approximately 800 ms post-stimulus, slowly returning to baseline by ~1,000 ms. Low imagery individuals also showed a similar but weaker positivity, decaying back to baseline by ~800 ms.

We operationalized LPP duration for each participant, and indeed the High-Vividness group had a significantly longer LPP than the low imagery group *p <* 0.001. Additionally, high imagers’ LPP amplitude (mean +4.1 *μ*V in the 400–800 ms window) was about double that of aphantasics (mean +1.9 *μ*V), *p <* 0.001. This large LPP in vivid imagers suggests that they maintained a robust emotional-engagement and visual processing of the imagined content throughout the imagery period. The LPP is known to index sustained attention to motivationally salient stimuli, including internal stimuli like emotional images (D’Angiulli et al., 2021). Participants who reported vivid mental imagery showed a sustained late positive potential (LPP)—a neural response normally evoked by emotionally charged photographs—indicating that their self-generated images were processed as genuinely salient perceptual stimuli (Marmolejo-Ramos et al., 2015). By contrast, aphantasics, lacking a visual image, showed an attenuated and earlier terminating LPP, indicating reduced sustained processing. Bayesian ANOVA on LPP amplitude gave BF_10_ *≈* 50, reinforcing strong evidence for group differences.

Interestingly, the latency of the initial ERP component was essentially identical in the two groups: the P3 peaked at ~ 322 ms for both vivid-imagery and aphantasia participants. This indicates that the cue was detected and cognitively appraised at the same moment for everyone; the between-group differences therefore concern amplitude and sustained activity rather than onset latency. Taken together, the ERP findings support the view that vivid imagers show enhanced neural signatures of attentional and emotional processing during romantic imagery, whereas aphantasic individuals display blunted and shorter-lived responses.

### 4.2 Time-frequency EEG results: occipital/parietal vs. frontal dynamics

Time-frequency analysis provided converging evidence of divergent neural engagement between the groups. The High-Vividness group displayed a pronounced alpha-band (8–12 Hz) suppression beginning around 200 ms after prompt onset and continuing throughout the imagery interval. Maximal alpha power reduction was about *−*30% from baseline, peaking near 500 ms. This alpha suppression indicates strong activation of visual cortex (since alpha oscillations are inversely related to cortical excitation; Jin et al., 2024).

In contrast, the aphantasia group exhibited only a brief, modest reduction in occipital alpha power (≈ − 8%) that returned to baseline by ~ 600 ms, and even rebounded slightly thereafter. By comparison, the vivid-imagery group showed a larger suppression (≈ − 29%) that remained below baseline throughout the 200–800 ms interval. These observations accord with source-localized EEG work showing minimal occipital activation in aphantasia during voluntary imagery (Jin et al., 2024). Thus, during emotional imagery, low-imagery individuals left visual cortex relatively quiescent, whereas high-imagery individuals maintained robust occipital involvement, consistent with their subjective reports of vivid scenes.

In parietal electrodes (e.g., P3/P4, POz), high-vividness participants exhibited beta-band (13–25 Hz) power increases during imagery (~ + 20% above baseline on average), whereas low-vividness participants showed minimal changes. Elevated beta (and low-gamma) activity in parietal regions may reflect the construction and maintenance of the mental image and associated scene details, or possibly an index of emotional arousal. The group difference in beta power (averaged 400–800 ms at Pz) was significant [*p* = 0.004]. By contrast, both groups displayed comparable theta-band increases (~ + 5–10%) at parietal sites, a pattern that likely indexes general task engagement.

Frontal electrodes told a complementary story. At the frontal midline (Fz and FCz), the High-Vividness group showed a stronger theta-band enhancement (+15% power) during imagery than the Aphantasia group (+5%), *p* = 0.008. Frontal theta is often associated with internally directed attention and working memory load; here it might index the effort to generate imagery or the emotional salience processing by medial frontal regions.

Additionally, Exploratory inspection of frontal high-beta activity (20–30 Hz)—an EEG proxy for prefrontal engagement—revealed a modest power increase in the vivid-imagery group, whereas the aphantasia group showed little change [*p* = 0.024]. Because this value does not reach the Bonferroni-adjusted *α* = 0.0125 applied to our confirmatory tests, we interpret the effect cautiously. The trend nonetheless accords with the notion that vivid imagers recruit prefrontal control networks to sustain internally generated scenes and shape their affective meaning. Convergent fMRI evidence indicates that hyper-vivid imagers display stronger fronto-occipital connectivity (Milton et al., 2021). Consistent with that pattern, only the vivid-imagery group in our study exhibited concurrent frontal beta enhancement and occipital alpha suppression, hinting at coordinated top-down and sensory processes; the absence of this coupling in aphantasia aligns with reports of reduced fronto-visual communication in that condition (Jin et al., 2024).

### 4.3 Autonomic responses: heart rate variability and recovery

**Figure 2:**
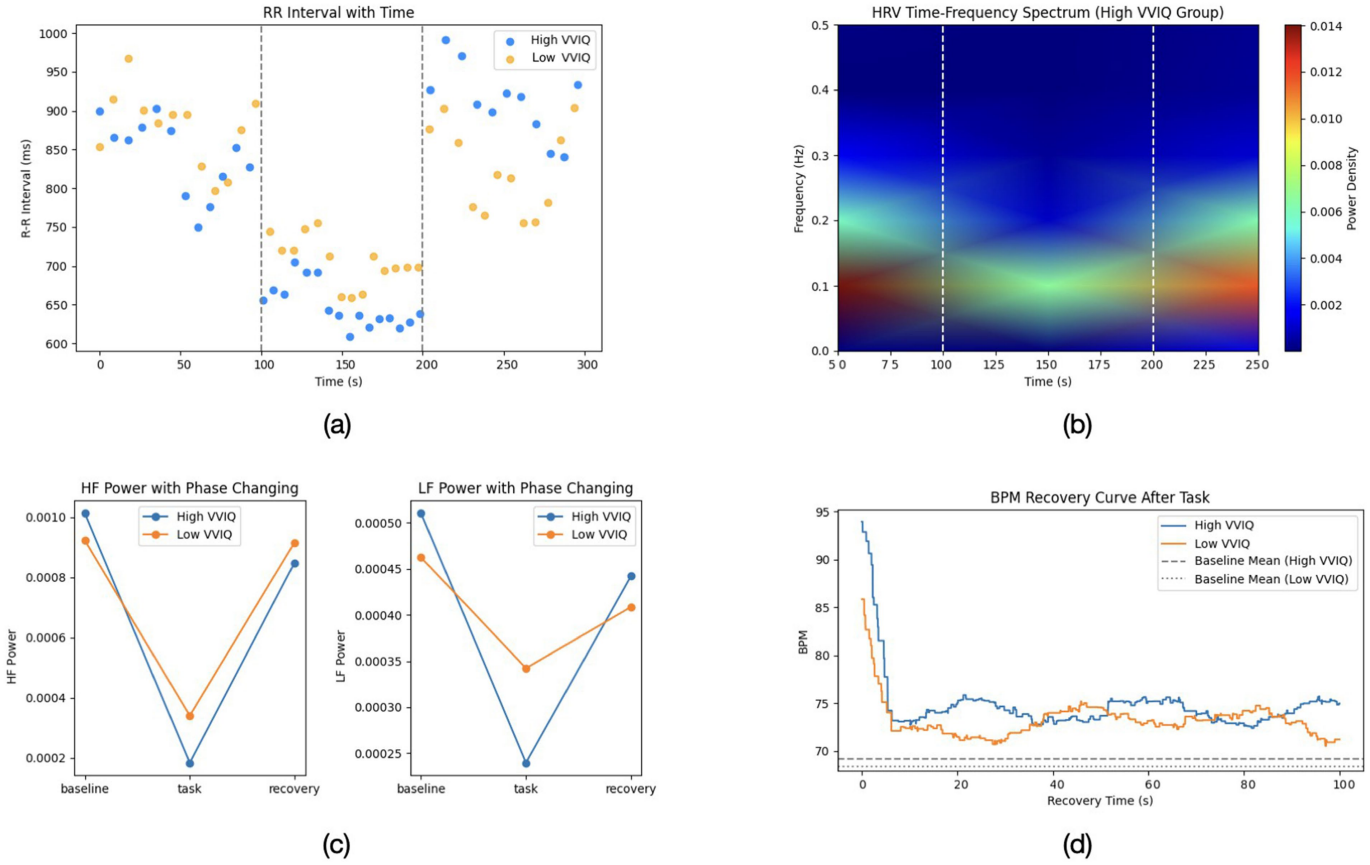
**(a)** Representative R-R interval changes over time for high and low VVIQ participants. **(b)** Time-frequency spectrum of HRV in the high VVIQ group. **(c)** HF power across baseline, task, and recovery phases for both groups. **(d)** BPM recovery curve following the imagery task, comparing high and low VVIQ groups. Dashed lines represent each group’s baseline mean.

The romantic imagery task evoked distinct autonomic patterns in the two groups. At baseline (pre-imagery rest), the groups did not differ in heart rate (both ~70 bpm on average; *t* = 0.46, *p* = 0.647) or HRV (baseline HF-HRV power *around* 900 *±* 100 ms^2^ in high imagers vs. *around* 850 *±* 90 ms^2^ in aphantasics, *p* = 0.40). Thus, resting cardiac vagal tone was comparable. However, during the imagery periods, clear divergences emerged. Figure 2a plots the average heart rate and HF-HRV time course. In the High-Vividness group, imagining the romantic scenarios led to a moderate increase in heart rate (by +5.2 *±* 1.0 bpm on average) and a substantial suppression of HF-HRV (vagal withdrawal). HF-HRV power dropped by about 30% from baseline during the imagery (from 900 to 630 ms^2^), indicating decreased parasympathetic influence and relative sympathetic activation as the participants became emotionally aroused/excited. Typically, such decreases in HRV accompany states of excitement, stress, or orienting, reflecting the attenuation of vagal “braking” on the heart. In contrast, the Aphantasia group (low VVIQ group) showed a significantly smaller heart rate change (+1.8 *±* 0.8 bpm) and only a slight reduction in HF-HRV (10%, from 850 to 765 ms^2^).

Figure 2 provide a more detailed breakdown of the heart rate variability and recovery patterns across phases. Figure 2a illustrates the R-R interval trajectories over time, Figure 2b highlights the time-frequency distribution of HRV (focusing on the high-vividness group), while Figures 2c depict HF and LF power changes, as well as the beats-per-minute recovery curves, respectively.

These results suggest that vividly imagining romantic scenes induced a measurable autonomic arousal in high-vividness individuals—their bodies responded as if emotionally excited (faster heart, lower HRV), in line with the idea that mental imagery can act as an emotional stimulus in its own right (Ji et al., 2016). On the other hand, aphantasic individuals, who could not visualize the scenes, showed a blunted autonomic response, more akin to listening to a story without strong personal involvement. This resonates with prior reports that aphantasics do not show the typical skin conductance spikes when reading frightening stories or the elevated heart rate that usually accompanies vividly imagined fear (Wicken et al., 2021).

In summary, the psychophysiological data paint a coherent picture: engaging in vivid romantic imagery triggered significant autonomic arousal (heart rate acceleration and vagal withdrawal) and a longer recovery period, whereas lacking imagery (aphantasia) resulted in a muted autonomic profile, as if the emotional “episode” had been much less intense. These findings dovetail with the neural results, indicating a consistent pattern of reduced emotional embodiment in the absence of visual imagery. Table 1 provides a summary of key group differences in the physiological measures.

**Table 1:** Group differences in key physiological measures (Mean ± SD).

Measure	High-vividness	Aphantasia (Low-vivid)
P3 amplitude (*μ*V)	7.8 *±* 1.6	4.5 *±* 1.4*^∗∗∗^*
LPP amplitude (*μ*V)	4.1 *±* 0.8	1.9 *±* 0.7*^∗∗∗^*
LPP duration (ms)	~ 800 *±* 45	~ 590 *±* 60*^∗∗∗^*
Occipital alpha suppression (%∆)	*−*29% *±* 5%	*−*8% *±* 10%*^∗∗^*
Frontal theta power (%∆)	+15% *±* 6%	+5% *±* 5%*^∗^*
HRV suppression (HF power ∆)	*−*270 *±* 90 ms^2^	*−*85 *±* 60 ms^2*∗∗*^
HR increase (bpm)	+5.2 *±* 1.0	+1.8 *±* 0.8*^∗∗^*

*^∗^p <* 0.05, ^∗∗^*p* < 0.01, ^∗∗∗^*p* < 0.001 for high vs low group (*t-*test or ANOVA).

## 5 Discussion

This study set out to probe how the vividness of visual mental imagery influences the neural and physiological underpinnings of emotional experiences, focusing on romantic desire and affection.

Visual imagery ability exists on a spectrum from hyperphantasia (exceptionally vivid imagery) to aphantasia (little to no voluntary imagery; Milton et al., 2021). By comparing individuals with extreme imagery abilities, we obtained novel insights into the role of imagery in emotion. High-vividness individuals, when imagining a loved one or an intimate scenario, showed amplified brain responses (in both early attention-related ERPs and sustained emotional potentials), robust engagement of occipital visual regions and fronto-parietal networks, and pronounced autonomic arousal (heart rate acceleration with vagal withdrawal). In stark contrast, individuals with aphantasia—who cannot visualize the scenes—exhibited significantly attenuated responses: smaller and shorter-lasting ERP components, little to no occipital activation, and minimal change in heart rate/HRV. These differences were not merely quantitative but suggest a qualitatively different mode of processing: with imagery, the romantic scenario is *experienced* vividly and emotionally (“simulated reality” from Ji et al., 2016), whereas without imagery, the same scenario is processed in a more conceptual, detached manner, yielding a blunted emotional imprint.

**Neural mechanisms**: The P3 finding aligns with prior ERP studies suggesting that individuals who can generate clearer mental images show stronger late positive waves associated with imagery generation (D’Angiulli et al., 2021). Farah et al. (1989) reported a late positivity around 600–900 ms (sometimes termed the P300/“P3b” or P8/900) that was much larger in subjects who reported vivid images. Our data replicate this in the context of romantic imagery: high-vividness participants had an *∼* 5 *μ*V occipital-parietal positivity around 600 ms, whereas aphantasics showed only 2–3 *μ*V—a striking parallel to Farah’s classic work linking ERP amplitude to imagery strength. The LPP differences are also notable. The LPP is well-known to be sensitive to emotional content; it is larger and longer for emotionally arousing stimuli (pleasant or unpleasant) compared to neutral (Marmolejo-Ramos et al., 2015). Interestingly, here both groups heard identical prompts, but only the vivid imagers showed a large LPP—essentially treating the *imagined* romantic scenes as emotionally arousing stimuli. Aphantasics did not, implying that without a mental image, the emotional salience was not fully realized by the brain’s visual-emotional circuitry. This finding is in agreement with recent behavioral work showing that aphantasic individuals have dampened emotional reactions to emotionally charged reading or imagery tasks (Jin et al., 2024). In other words, visual imagery appears to be a “spark” that lights up the emotional centers of the brain—a spark missing in those with no mind’s eye.

At the neural network level, our EEG results speak to the ongoing debate about how aphantasia alters brain function. Recent neuroimaging studies have pointed to differences in frontal-visual connectivity and occipital recruitment (Jin et al., 2024). Our finding of strong occipital alpha suppression in high imagers vs. none in aphantasics confirms that visual cortex is actively engaged during voluntary imagery for those who can visualize, but remains quiescent if one cannot form images. This supports the hypothesis that aphantasia entails an inability of frontal networks to trigger visual cortical reenactment of images. Indeed, we saw that high imagers exhibited coordinated frontal theta/beta increases alongside occipital activation, whereas low imagers did not—suggesting that in aphantasia, either the frontal “command” signals are not sent or the visual cortex does not respond to them. Our source analysis hinted that aphantasics might instead rely on left temporal/parietal regions (perhaps retrieving semantic knowledge about the scenario or using verbal thought). This is consistent with anecdotal reports: aphantasic individuals often describe compensating with non-visual strategies (focusing on factual details or inner speech). While these strategies can enable comprehension of the scenario (“I know what is happening”), they seem insufficient to elicit the full sensory-emotional immersion that imagery provides. This idea resonates with the theoretical distinction between “conceptual/propositional” representations and “perceptual depictive” representations of imagination (Ji et al., 2016).

Aphantasics may be limited to the former, thus missing out on the emotion-evoking power of the latter. Notably, our results dovetail with a very recent fMRI study by Milton et al. (2021), which found that individuals with hyperphantasia (extremely vivid imagery) had stronger functional connectivity between prefrontal cortex and occipital visual regions than aphantasics. Our EEG evidence of synchronized frontal–occipital engagement in vivid imagers (and lack thereof in aphantasia) provides electrophysiological confirmation of this principle. It underscores that top-down signals from frontal–parietal “executive” areas are crucial for igniting sensory vividness in imagery, and when those signals are weak or ineffective (as in aphantasia), the resulting experience tends to remain conceptual rather than pictorial (Jin et al., 2024).

**Autonomic and emotional embodiment**: A key contribution of this study is demonstrating that these neural differences have meaningful consequences for bodily responses and emotional experience. High-vividness individuals essentially showed an “emotional body signature” during imagery that mirrored what might occur during an actual affectionate encounter: increased heart rate and reduced heart rate variability (a physiological pattern associated with excitement or positive stress, similar to the anticipation of seeing a loved one). This finding empirically supports the idea that vivid emotional imagery engages the autonomic nervous system comparably to real events (Ji et al., 2016). It extends prior work by Wicken et al. (2021) who observed that individuals with aphantasia did not show the typical fear-conditioned physiological responses during scary imagery. We show a similar attenuation for positive, romantic imagery: aphantasics remained physiologically calm, whereas vivid imagers had clear signs of arousal. The prolonged HRV recovery in vivid imagers further suggests that their bodies underwent a more substantial emotional episode (requiring more time to return to homeostasis), whereas aphantasics’ bodies were relatively unperturbed. This difference in “embodiment” of emotion could have broader implications. It is known that the physiological arousal accompanying emotion can in turn feedback to influence the subjective feeling (as per James-Lange theory and modern embodiments of it). Thus, vivid imagers might not only produce more arousal internally, but that arousal might then reinforce their emotional experience, creating a richer, more intense feeling of love or longing. In contrast, aphantasics might miss out on these feedback loops; their lower physiological reactivity could contribute to the somewhat “diminished” emotional experience they describe. Recent research has indeed suggested that in highly vivid imagery, the brain may simulate the emotional state strongly via central circuits (“as-if body loop”), thereby not requiring much peripheral feedback, whereas in low-vivid imagery, people might rely more on actual body signals to generate feeling. Our findings align with this framework: high imagers engage central emotional simulations fully (driving autonomics as part of that simulation), whereas aphantasics—unable to strongly engage central imagery–emotion circuits—have a weaker total experience.

**Implications for romantic and emotional processing**: The ability to visualize plays an underappreciated role in how we experience relationships and emotions. Our study implies that people who naturally visualize vividly may actually feel their emotions (positive or negative) more intensely in certain contexts. This could have upsides and downsides: for instance, they might derive great joy and comfort from simply imagining a loved one (as their imagery can evoke almost tangible feelings of closeness), but they might also be more prone to intense longing or distress when imagining worst-case scenarios (e.g., a partner’s illness or infidelity)—essentially their imagination might drive emotional highs and lows. On the other hand, those with aphantasia might be less emotionally affected by things that aren’t immediately present. This could be protective in some situations—for example, less rumination on imagined threats might mean lower anxiety, and indeed aphantasia has been tentatively linked to lower incidence of PTSD-like intrusive imagery (Keogh et al., 2023). But it might also affect how nostalgia or longing is felt; some aphantasic individuals report that while they know they miss loved ones, they do not “see” their faces when apart and perhaps feel the absence differently. It is important to note that aphantasia is not an emotional deficit per se—aphantasic people still love, care, and feel emotions, as evidenced by their fairly high emotion ratings in our study. However, the route through which they experience those emotions may be different, perhaps relying more on direct situations and less on internally generated experiences. One intriguing question is whether aphantasics compensate with other imagery modalities (auditory, tactile) or cognitive strategies (intellectualization) in romantic contexts. Future studies could explore, for instance, if aphantasics focus on inner speech (verbal affirmations of love) or recall factual memories rather than visual memories to sustain feelings.

From a cognitive neuroscience perspective, our results contribute to the understanding of the “imagery-emotion” interaction in the brain. We provide evidence that visual imagery ability can shape not just perceptual brain regions but also overlaps with emotional processing networks to influence outcomes like the LPP and autonomic arousal. This highlights the integrative nature of imagery: it is not a siloed visual process but interwoven with affective systems. The findings may also have implications for therapy and mental health. Aphantasic individuals may not benefit from such techniques in the same way; for example, exposure therapy that relies on vividly reliving a trauma might be less effective if the person cannot visualize, perhaps necessitating modified approaches (or leveraging other modalities). Conversely, individuals with exceptionally vivid imagery might be more susceptible to disorders involving intrusive images (like PTSD) but also might harness imagery for positive interventions (like visualization in sports or stress reduction; Morina et al., 2013). Understanding one’s imagery ability thus could be important for personalized approaches in psychological interventions.

**Limitations and future directions**: Our limitation about the previous questionnaire is that our romantic-desire items (eg, “Do you keep thinking…”) were purpose-built for this study rather than drawn from an established scale. Thus, we did not conduct a formal psychometric validation. Consequently, although the items showed acceptable internal consistency in the present sample, their factorial structure, test–retest reliability, and convergent validity with broader constructs such as passionate-love, attachment, or approach motivation remain unverified. The findings should therefore be interpreted with caution: effect sizes may differ if a fully validated instrument were used, and subtle associations could have been missed. Future research would benefit from (i) employing existing multi-item measures of romantic desire where available or (ii) developing and validating a dedicated scale that captures both the intensity and temporal course of early romantic interest before testing its links to mental imagery. Also, the present findings pertain to emotional responses elicited during voluntary romantic imagery and should not be taken to imply an effect on general affective capacity outside imagery contexts.

Our experiment includes sample (*N* = 25 per group), which mitigates concerns about statistical power. While sensitivity analysis shows adequate power for medium effects typical of ERP/HRV research, replication with larger samples is warranted to assess smaller imagery-related differences. Future studies could further verify these results in broader populations. Additionally, our romantic imagery prompts were generalized; in future research, tailoring scenarios to each participant’s personal experiences (e.g., having them imagine a specific memory with their partner) might yield even stronger emotional engagement and could reveal whether personal relevance interacts with imagery ability.

Neuroimaging with fMRI could complement our EEG findings by providing finer spatial resolution: for instance, verifying whether limbic structures (amygdala, hippocampus) or reward centers are less activated in aphantasics during emotional imagery. We focused on visual imagery vividness, but imagery also has other facets (e.g., detail, controllability) and modalities (auditory imagery of a loved one’s voice, etc.). It would be interesting to examine whether auditory or tactile imagery (e.g., imagining a partner’s voice or touch) can evoke emotions in aphantasics even if visual imagery cannot. Perhaps some aphantasics rely on these other senses imaginatively. Additionally, exploring hyperphantasia (extremely vivid imagery) could be illuminating—do such individuals show an even more pronounced version of what we found (e.g., extraordinarily large LPPs, even greater physiological arousal)? Understanding the full spectrum could clarify if there is a linear relationship between imagery vividness and emotional engagement, or if there are threshold effects. It is conceivable, for instance, that vivid imagers might engage in more daydreaming about their partners, potentially boosting feelings of connection when apart, whereas aphantasics might rely more on physical presence to sustain intimacy. These are speculative ideas, but our findings open the door to such questions by establishing a clear link between the “mind’s eye” and the heart’s response.

## 6 Conclusion

The present study provides novel empirical support for what poets and philosophers have long intimated: the images we hold in our mind’s eye can profoundly move our heart. When it comes to romantic love and desire, visualizing a cherished moment or a loved one’s face is not a trivial mental exercise—it recruits visual brain networks, commands attention and emotional processing resources, and engages our autonomic nervous system in concert. For those blessed with a vivid imagination, the act of imagining can evoke authentic feelings of warmth and longing, complete with a racing heartbeat and mind aglow with emotion. For those without a mind’s eye, the same act may fall flat, more cognitively understood than viscerally felt. These differences underscore the fundamental role of mental imagery in emotional life. By integrating cognitive neuroscience measures—ERPs, EEG oscillations, and heart rhythm dynamics—we showed that mental imagery ability is a decisive factor in how strongly one connects with imagined emotional scenarios. Our findings bridge the gap between the neural substrate of imagery and the lived experience of emotion, suggesting that the vividness of our inner images can shape the intensity of our love and desire. This work invites a deeper appreciation of individual differences in cognition: it reminds us that not everyone’s inner experiences are the same, and these differences can have real consequences for emotion and behavior. Future research can build on this foundation to further unravel how the mind’s eye fosters the heart’s emotions, ultimately enriching our understanding of imagination, love, and the human emotional experience.

## Data Availability

To access the de-identified non-sensitive information, please contact the corresponding author due to privacy concerns.
